# Nomogram for predicting amputation-free survival in acute lower limb ischemia patients treated by endovascular therapy

**DOI:** 10.1016/j.heliyon.2024.e32110

**Published:** 2024-05-29

**Authors:** Hao Huang, Jie Kong, Xu He, Liang Chen, Haobo Su

**Affiliations:** Department of Interventional Radiology, Nanjing First Hospital, Nanjing Medical University, Nanjing, China

**Keywords:** Acute lower limb ischemia, Amputation-free survival, Risk factors, Prediction, Nomogram

## Abstract

**Objectives:**

To develop a novel and accurate nomogram to predict survival without amputation in patients with acute lower limb ischemia (ALLI) during the first year following endovascular therapy.

**Methods:**

Patients with ALLI who underwent endovascular therapy in our department between January 2012 and September 2020 were screened and included in the research. The included patients were randomly divided into a training and validation cohorts, respectively. Univariate and multivariate analyses were used in the training cohort to identify independent risk factors for amputation-free survival (AFS). A nomogram was then developed according to the identified independent risk factors. The nomogram was then validated in the validation cohort.

**Results:**

415 Chinese patients with 417 affected limbs were included in this study. Among these patients, 311 patients were classified into the training cohort and 104 patients were assigned to the validation cohort. Most patients were men (n = 240) and the average age of patients was 71.43 (standard deviation 8.86) years old. After the univariate and multivariate analyses, advanced age (*p* < 0.001), history of smoking (*p* < 0.001), atrial fibrillation (*p* < 0.001), and insufficient outflow (*p* = 0.001) were revealed as independent risk factors for AFS during the first year. The nomogram yielded AUROC values of 0.912 (95 % confidence interval [CI]: 0.873–0.950) and 0.889 (95 % CI: 0.812–0.967) in the training and validation cohorts, respectively.

**Conclusion:**

Advanced age, history of smoking, atrial fibrillation, and insufficient outflow were independent negative predictors for AFS in ALLI patients treated by endovascular therapy. The novel nomogram offered an accurate prediction of AFS in ALLI patients.

## Strengths and limitations of this study

1 Offered an accurate prediction of AFS in ALLI patients.
2 This was a retrospective study.
3 Prospective cohorts in other settings were needed.

## 1 Introduction

Acute lower limb ischemia (ALLI) is characterized by a sudden and rapid decrease in perfusion to the lower extremities, posing a serious threat to their viability [1]. An acute condition is defined as symptoms that last for <14 days [2]. At present, arterial thrombosis, predominantly associated with atherosclerosis (AS), serves as the primary cause of ALLI [3]. In contrast to chronic limb ischemia (CLI), in ALLI, there is insufficient time for angiogenesis to make up for perfusion loss ^3 4^. Thus, immediate recognition and emergency revascularization should be performed to preserve limb viability [5]. It is important to diagnose and assess the disease early, although misdiagnosis can heighten amputation and mortality rates, despite significant progress in the treatment of blood disorders [6]. Endovascular therapy is one of the most commonly used treatments [27], and amputation or death is the most severe outcome for ALLI cases [[2], [3], [4], [5]]. Amputation-free survival (AFS) is the primary treatment goal, as it can improve patients' quality of life in the future. However, the types of patients who clearly benefit from endovascular therapy have not been thoroughly studied. With the easy accessibility and accurate risk assessments based on significant factors, nomograms have been increasingly utilized to predict specific clinical outcomes for individual patients. Although a nomogram can be a helpful tool for assisting medical professionals in determining diagnostic and therapeutic strategies for various diseases, it has not yet been used for predicting ALLI [8,9]. Predicting a patient's possibility of AFS can help patients and their families make better choices about treatment and patient care. The present study aimed to investigate the first year outcomes of endovascular therapy in patients with ALLI. Then, the study aimed to establish and validate an effective nomogram that can accurately predict survival without amputation during the year following endovascular therapy. This would help clinical staff identify patients at higher risk and ensure they receive timely appropriate therapies.

## 2 Design and methods

### 2.1 Patient criteria

The retrospective study was granted approval by the Ethical Review Committee of Nanjing First Hospital. All procedures were conducted in accordance with approved guidelines established by the ethics committee and adhered to the principles outlined in the Declaration of Helsinki. The retrospective nature of the study resulted in a waiver of informed consent. Between January 2012 and September 2020, consecutive symptomatic ALLI cases who underwent endovascular therapy treatment were screened and enrolled. We considered a symptom duration of ≤14 days as the maximal time threshold [10]. According to the randomized 3:1 allocation procedure, three-fourths of the patients were placed in the training group. This group was used to develop a predictive nomogram model. The remaining patients were assigned to the validation group, which was used to assess the performance of the nomogram model.

Patients meeting the following inclusion criteria were enrolled in this study [2,11]: evidence of occlusion of the lower limb artery on computed tomographic angiography (CTA) or ultrasound; aged 18 years or older; symptom duration of 14 days or less; and acute ischemia classes I-IIb, according to the acute Rutherford classification for ALLI. Patients who were ineligible for anticoagulants, antiplatelet therapy, or thrombolytic drugs; those with current malignant disease; individuals with a history of a life-threatening reaction to contrast medium; patients who had experienced cerebral bleeding or ischemic stroke within the past 6 weeks; or those who had undergone surgery within the past 6 weeks were excluded from the study.

### 2.2 Patient and public involvement

This research did not involve any participation or input from the general public or patients in terms of the study's design, implementation, reporting, or dissemination plans. Thrombolysis protocols and anticoagulant and antiplatelet therapy.

Each procedure was performed with local anesthesia through either the femoral artery on the same side as the affected area or using an approach from the opposite side. Subsequently, a multi-hole infusion catheter (Medtronic, Minneapolis, USA) was inserted into the thrombus, followed by administering a 4-mg bolus of recombinant tissue plasminogen activator (Actilyse, Boehringer, Sweden), which was then infused at a dose of 0.55 mg/h. Lower limb angiograms were obtained at intervals of 24 (±4) h as a control. The initial angiographic study was carried out early in cases whose catheter-directed thrombolysis (CDT) was initiated by the end of the day. For lytic treatment, outcomes included flow restoration and complete lysis. Plain radiography and duplex ultrasound were adopted as necessary to differentially diagnose the residual thrombus during the process of CDT. We monitored blood test results a minimum of once per day (including complete blood count, sodium, potassium, myoglobin, creatinine, and creatine kinase content). Other tests were also conducted if needed. Fibrinogen content was observed at least once per day. Essential endovascular therapy (i.e., stenting or percutaneous transluminal angioplasty [PTA]) or endovascular thrombectomy (e.g. Angiojet device or Straub Rotarex system) was applied for complete thrombolysis to restore the bypass graft or vessel patency and achieve sufficient distal perfusion. In addition, each patient was given a 40-mg subcutaneous injection of low-molecular-weight heparin enoxaparin sodium (Klexane; Sanofi-Aventis, France) twice a day for various durations (from days to weeks) depending on the need for continuous oral anticoagulant treatment and the physical activity level of the patients. Additionally, for cases that showed AS changes that could potentially result in thrombotic occlusions, 100 mg of aspirin was administered. In patients receiving femoropopliteal segment PTA, clopidogrel at 75 mg/day was administered alone or with aspirin post-procedurally for at least 6 months. For cases showing atrial fibrillation (AF), cardiac thrombus, occlusions, or an unclear cause, and hyper-coagulability, warfarin or new analogs were administered for varying durations or enduringly. For these patients, individualized decisions were made, and in some patients, the above medications were combined.

### 2.3 Follow-up

The follow-up period was defined as 1 year or the date at which major amputation or mortality occurred within the first year. We also accounted for thromboembolic events in identical vascular segments or vessels or bypass grafts. Each case was monitored for different durations. Most patients underwent the initial follow-up at 1year post-procedurally. All risk factors reported in the guidelines and literature will be recorder [3,7,[12], [13], [14], [15], [16]]. Before the treatment, we collected demographic data (i.e., sex and age), duration between symptom occurrence and admission, clinical presentation (e.g., sensory loss, pain, reduced skin temperature, and motor impairment), concurrent diseases, and risk factors (e.g., hyperlipidemia, hypertension, AF, and smoking history) of patients. We also collected thrombosis-associated data (i.e., location and length), which were obtained by angiogram and/or CTAs. The therapeutic strategy for every case (option of balloon catheter, stent, time to lysis, and antithrombotic agent use, which included anticoagulant or antiplatelet agents), alongside procedure-associated data (technical success and procedure-related complications, such as procedure-associated early reintervention, distal embolization, or aneurysm formation/rupture), was recorded. The primary outcome for the analysis was the occurrence of amputation or death during the first year following treatment. Follow-up outcomes (e.g., amputation and mortality) were recorded and analyzed.

### 2.4 Statistical analysis

The continuous variables are presented as means ± standard deviations, while the categorical variables are displayed as percentages. Univariate regression analysis was conducted to identify potential risk factors. Subsequently, variables that showed a significant association with a AFS(p < 0.05) were included in the subsequent multivariate regression analysis. The rms package of R software (version 3.0.2; https://www.r-project.org/) was utilized to construct a nomogram based on the results of the multivariate analysis. A p-value less than 0.05 indicated statistical significance. Statistical analyses for identifying risk factors were performed using SPSS 18.0 for Windows (IBM Corporation, Somers, NY, USA).

## 3 Results

### 3.1 Clinical characteristics of the study cohort

From January 2012 and September 2020, 415 patients were enrolled, which comprised 417 affected limbs. 311 patients were divided into the training cohort for developing a predictive nomogram model, while 104 patients were assigned to the validation cohort for evaluating the model's performance. Most cases were men (n = 240, 57.8 %), and the mean age of patients was 71.43 (8.86) years. Of the 417 treated limbs, 186 (44.6 %) were right limbs, and 231 (55.4 %) were left limbs. The mean duration of ischemia was 67.15 (53.54) hours. During the follow-up period, a total of 37 (11.8 %) limbs in the training cohort and 18 (17.3 %) limbs in the validation cohort underwent major amputations. In the two cohorts: 233 of 311 (74.9 %) patients in the training cohort were confirmed with AFS and 69 of 104 (66.3 %) patients were confirmed in the validation cohort. There were no significant differences (P > 0.05) between the two cohorts in terms of basic clinical characteristics. Details of patients' features are provided in Table 1.

**Table 1:** Patient characteristics.

	Training cohort (n = 311)	Validation cohort (n = 104)	*P*-value
**No. patients**	311	104	
**Sex**			0.731
Male	178 (57.2 %)	62 (59.6 %)	
Female	133 (42.9 %)	42 (40.4 %)	
**Age (years)**	71.59 (8.94)	70.93 (8.64)	0.503
**Limbs**	313	104	0.255
Left	168 (53.7 %)	63 (60.6 %)	
Right	145 (46.3 %)	41 (39.4 %)	
**Comorbidities**			
Diabetes	120 (38.6 %)	36 (34.6 %)	0.486
Arterial hypertension	242 (77.8 %)	71 (68.3 %)	0.065
Coronary heart disease	42 (13.5 %)	16 (15.4 %)	0.627
Cerebrovascular disease	57 (18.3 %)	16 (15.4 %)	0.554
Renal insufficiency	23 (7.4 %)	4 (3.8 %)	0.255
Atrial fibrillation	227 (73.0 %)	76 (73.1 %)	1.000
**History of smoking**	94 (30.2 %)	34 (32.7 %)	0.626
**Duration of ischemia (hours)**	67.24（54.88）	66.90（49.57）	0.316
**Type of occluded vessel**			1.000
Native artery	308 (98.4 %)	103 (99.0 %)	
Bypass grafts	5 (1.6 %)	1 (1.0 %)	
**Ischemia Rutherford category**			0.696
I	13 (4.2 %)	4 (3.8 %)	
IIa	106 (33.9 %)	40 (38.5 %)	
IIb	194 (62.0 %)	60 (57.7 %)	
**Proximal segment involved**			0.813
Iliac	17 (5.4 %)	4 (3.8 %)	
Femoral	75 (24.0 %)	25 (24.0 %)	
Popliteal-tibial	221 (70.6 %)	75 (72.1 %)	
**PTA/Stent**	109 (34.8 %)	38 (36.5 %)	0.813
**Endovascular Thrombectomy**	31 (9.9 %)	9 (8.7 %)	0.848
**Amputation**	37 (11.8 %)	18 (17.3 %)	0.180
**Amputation-free Survival**	233 (74.9 %)	69 (66.3 %)	0.099

PTA = percutaneous transluminal angioplasty.

### 3.2 Construction of the predictive nomogram for ALLI in patients with AFS

**Fig. 1:**
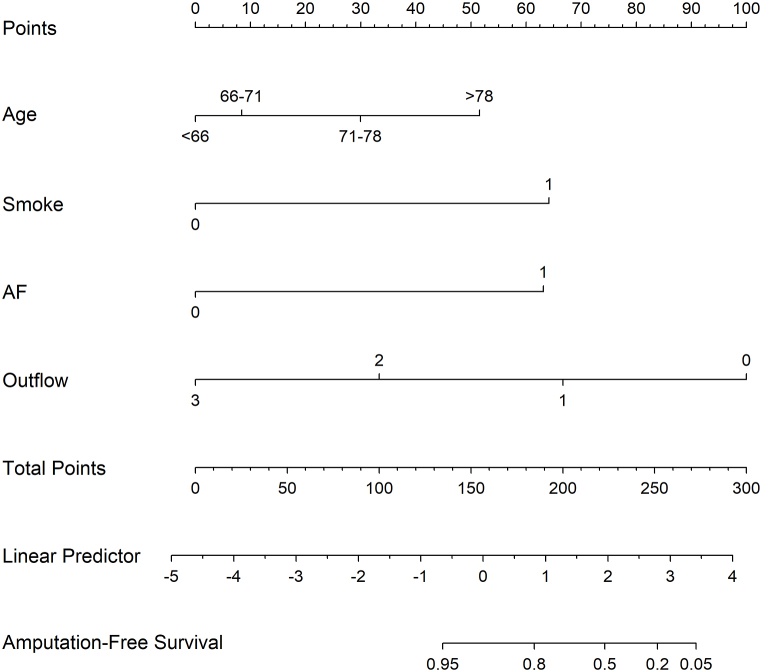
Nomogram to predict amputation-free survival (AFS) following endovascular treatment in patients with acute lower limb ischemia. To use the nomogram, an individual patient's total score is located on each variable axis, and a line is drawn upward to determine the number of points for each variable. The sum of these points is located on the Total Points axis, and a line is drawn downward to the probability axes to determine the probability of AFS.

In the training cohort, Table 2 shows the radiological and clinical data of patients in the AFS and non-AFS groups. In the univariate analysis, advanced age, Rutherford category, history of smoking, history of atrial fibrillation, and outflow number were predictors for AFS. Multivariate analyses revealed that age (*p* < 0.001), smoking history (*p* < 0.001), AF (*p* < 0.001), and insufficient outflow (*p* = 0.001) were independent predictive factors that predicted AFS within 1 year (Table 3). A nomogram for predicting AFS in ALLI patients was created using R software (Fig. 1) based on four independent risk predictors of AFS identified in the final multivariate logistic regression model. Each variable was assigned a score on the points scale. By adding up the scores and placing them on the total points scale, a vertical line could be drawn on the AFS scale to determine the predicted probability of AFS.

**Table 2:** Univariate analysis of predictors for amputation-free survival.

Characteristic	AFS	No AFS	HR	95 % CI	*P*-value
Sex					
Female	101	32	1		
Male	145	33	0.883	0.565–1.380	0.584
**Age (Years)**					0.024
1st quartile (<66)	63	11	1		
2nd quartile (66–71)	60	15	1.330	0.611–2.895	0.473
3rd quartile (72–78)	57	24	2.141	1.048–4.370	0.037
4th quartile (>78)	53	28	2.584	1.286–5.192	0.008
**Ischemia Rutherford Category**					0.063
I	10	4	1		
IIa	87	17	0.549	0.185–1.633	0.281
IIb	136	57	1.062	0.385–2.928	0.907
**History of smoking**					
No	192	25	1		
Yes	41	53	6.661	4.131–10.740	<0.001
**Atrial fibrillation**					
No	80	4	1		
Yes	153	74	8.074	2.951–22.087	<0.001
**Outflow**					<0.001
0	53	50	1		
1	111	25	0.301	0.186–0.487	<0.001
2	50	3	0.087	0.027–0.278	<0.001
3	19	0	0	0	0.951

*Cox regression analysis was used. The grouping of each parameter was based on statistical significance. AFS = amputation-free survival, HR = hazard ratio, CI = confidence interval.

**Table 3:** Multivariate analysis of the predictors for amputation-free survival.

Variable	B	HR	95 % CI	*P*-value
**Age (Years)**				<0.001
1st quartile (<66)	0	1		
2nd quartile (66–71)	0.202	1.223	0.557–2.687	0.616
3rd quartile (72–78)	0.868	2.383	1.163–4.884	0.018
4th quartile (>78)	1.457	4.294	2.099–8.783	<0.001
**History of smoking**	1.912	6.768	3.978–11.515	<0.001
**Atrial fibrillation**	1.826	6.211	2.253–17.120	<0.001
**Outflow**				0.001
0	0	1		
1	−0.705	0.488	0.293–0.833	0.008
2	−2.165	0.115	0.035–0.375	<0.001
3	−13.857	0	0	0.965

*Cox regression analysis was used. HR = hazard ratio, CI = confidence interval.

### 3.3 Validation of the predictive nomogram

Then, a verification of the nomogram model was completed in the training cohort and validation cohort. Calibration plots confirmed acceptable agreement between the actual and predicted AFS probabilities in the two cohorts. The nomogram yielded AUROC values of 0.912 (95 % confidence interval [CI]: 0.873–0.950) and 0.889 (95 % CI: 0.812–0.967) in the two cohorts.

## 4 Discussion

We established a simple nomogram for predicting the occurrence of AFS in ALLI patients treated by endovascular therapy. Several studies have identified the risk factors for AFS. However, results are inconsistent [15,17,18]. In this study, we identified age, history of smoking, AF, and insufficient outflow as independent negative predictors of AFS in ALLI patients. The most recent European Society for Vascular Surgery guidelines for the management of ALLI recommend catheter-directed thrombolysis as an alternative to surgery for patients with Rutherford grade IIa ALLI (recommendation 24, class I, level A). For patients with Rutherford grade IIb, prompt initiation of CDT may be considered and can be combined with percutaneous aspiration or thrombectomy (recommendation 25, class IIb, level B) [13].

Although thrombolysis has existed for several years, the role of insufficient outflow as a predictor remains under debate [2,4,[18], [19], [20], [21]]. Vakhitov and Byrne et al. reported that runoff vessel quantity predicts AFS [17,18]. However, Schrijver et al. reported the opposite result [15]. According to our work, runoff vessel number independently predicts AFS. The presence of runoff vessels reduces the formation of secondary thrombosis, and thus, the extremities receive blood more easily. As such, AFS occurs more often in the presence of sufficient runoff vessels.

In this work, advanced age and history of smoking were identified as factors that independently predicted AFS. Both these risk factors are consistently reported in previous studies. According to Grip and colleagues [6], age serves as a distinct risk factor for mortality (odds ratio 1.07/year), which is consistent with Vakhitov's findings (hazard ratio 5.23) [17]. Taha et al. and Schrijver et al. also reported similar results [17,19]. Moreover, observational studies have suggested that smoking is predictive of a greater chance of cardiovascular ischemic events, amputation, limb-related events and mortality [22,23]. However, age and smoking history are natural and unmodifiable factors. Thus, it is unsurprising that they have been consistently reported to predict death during the past 20 years [6,[24], [25], [26]].

This study demonstrated that AF is a risk factor for AFS. However, the role of AF in the death of patients who experience acute ischemia in the lower extremities after receiving CDT treatment remains unclear. AF may be related to the ineffectiveness of thrombolytic drugs on AF emboli. In addition, there are risk factors for AF, which usually coexist with congestive heart failure (CHF), and the co-occurrence of AF and CHF suggests that the patient has a higher mortality [27]. A comprehensive study conducted in Sweden encompassing over 200,000 cases experiencing incidental AF revealed that AF independently predicted all-cause mortality [22]. Moreover, in another large-scale research conducted by Benjamin et al. when the underlying AF-related cardiovascular disease was adjusted, AF increased the risk of death by 1.5–1.9 times [14].

To the best of our knowledge, the present work is the first study to attempt to estimate the probability of AFS in ALLI patients using a newly developed nomogram. Nomogram establishment is considered a feasible and credible approach for developing disease models [9,28]. In previous studies, determining the probability of AFS based on identified risk factors was not possible. Using this nomogram, it was possible to determine the probability of AFS simply yet accurately. Indeed, the nomogram analysis demonstrated that the probability of AFS was around 5 % among cases with no independent predictive factors and over 95 % among those with four independent predictive factors.

Such direct evaluations can help guide doctors to determine AFS. For patients with a low probability of AFS, surgical revascularization or percutaneous mechanical thrombectomy may be considered [5].

The study has several limitations. Firstly, this was a retrospective study, which has an inevitable risk of selection bias. Therefore, prospective studies are needed to validate the accuracy of the constructed nomogram and the risk factors using a more rigid method. Secondly, prospective cohorts in other settings require further study to validate the accuracy of our assessment tool.

In conclusion, this study demonstrated that advanced age, history of smoking, AF, and insufficient outflow were independent predictors of low AFS in ALLI patients. This novel constructed nomogram allowed objective and accurate predictions of AFS within 1 year of endovascular treatment. Further prospective investigations should be conducted to validate this nomogram.

## Ethics approval

The retrospective study was granted approval by the Ethical Review Committee of Nanjing First Hospital (2021080301, DATE:3rd, August 2021). All procedures were conducted in accordance with approved guidelines established by the ethics committee and adhered to the principles outlined in the Declaration of Helsinki.

## Funding

Project supported by the 10.13039/501100001809National Nature Science Foundation of China (No.81871463).

## Patient consent for publication

Not required.

## Data statement

The article and supplementary material encompass the original contributions made in this study. For any further inquiries, please contact the corresponding author.

## CRediT authorship contribution statement

**Hao Huang:** Writing – review & editing, Writing – original draft. **Jie Kong:** Software. **Xu He:** Supervision. **Liang Chen:** Supervision. **Haobo Su:** Writing – review & editing, Validation.

## Declaration of competing interest

The authors declare that they have no known competing financial interests or personal relationships that could have appeared to influence the work reported in this paper.

## References

[1] Norgren L., Hiatt W.R., Dormandy J.A. (2007). Inter-society consensus for the management of peripheral arterial disease (TASC II). Eur. J. Vasc. Endovasc. Surg..

[2] Creager M.A., Kaufman J.A., Conte M.S. (2012). Clinical practice. Acute limb ischemia. N. Engl. J. Med..

[3] Gilliland C., Shah J., Martin J.G. (2017). Acute limb ischemia. Tech. Vasc. Intervent. Radiol..

[4] van den Berg J.C. (2010). Thrombolysis for acute arterial occlusion. J. Vasc. Surg..

[5] Fluck F., Augustin A.M., Bley T. (2020). Current treatment options in acute limb ischemia. Röfo.

[6] Grip O., Wanhainen A., Acosta S. (2017). Long-term outcome after thrombolysis for acute lower limb ischaemia. Eur. J. Vasc. Endovasc. Surg..

[7] Theodoridis P.G., Davos C.H., Dodos I. (2018). Thrombolysis in acute lower limb ischemia: review of the current literature. Ann. Vasc. Surg..

[8] Hou G.M., Jiang C., Du J.P. (2021). Nomogram models for predicting risk and prognosis of newly diagnosed ovarian cancer patients with liver metastases - a large population-based real-world study. J. Cancer.

[9] O'Hagan L.A., Larsen P.D., Nataraja R.M. (2021). Nomogram of paediatric male urethral size: a systematic review. J. Pediatr. Urol..

[10] Investigators T.S. (1994). Results of a prospective randomized trial evaluating surgery versus thrombolysis for ischemia of the lower extremity. The STILE trial. Ann. Surg..

[11] Lian W.S., Das S.K., Hu X.X. (2020). Efficacy of intra-arterial catheter-directed thrombolysis for popliteal and infrapopliteal acute limb ischemia. J. Vasc. Surg..

[12] Zhang Y., Zhang Q.Q., Fu C. (2019). Clinical efficacy of tirofiban combined with a Solitaire stent in treating acute ischemic stroke. Braz. J. Med. Biol. Res..

[13] Bjorck M., Earnshaw J.J., Acosta S. (2020). Editor's choice - European society for vascular surgery (ESVS) 2020 clinical practice guidelines on the management of acute limb ischaemia. Eur. J. Vasc. Endovasc. Surg..

[14] Benjamin E.J., Wolf P.A., D'Agostino R.B. (1998). Impact of atrial fibrillation on the risk of death: the Framingham Heart Study. Circulation.

[15] Schrijver A.M., de Vries J.P., van den Heuvel D.A. (2016). Long-Term outcomes of catheter-directed thrombolysis for acute lower extremity occlusions of native arteries and prosthetic bypass grafts. Ann. Vasc. Surg..

[16] Piffaretti G., Angrisano A., Franchin M. (2018). Risk factors analysis of thromboembolectomy for acute thromboembolic lower extremity ischemia in native arteries. J. Cardiovasc. Surg..

[17] Vakhitov D., Oksala N., Saarinen E. (2019). Survival of patients and treatment-related outcome after intra-arterial thrombolysis for acute lower limb ischemia. Ann. Vasc. Surg..

[18] Byrne R.M., Taha A.G., Avgerinos E. (2014). Contemporary outcomes of endovascular interventions for acute limb ischemia. J. Vasc. Surg..

[19] Taha A.G., Byrne R.M., Avgerinos E.D. (2015). Comparative effectiveness of endovascular versus surgical revascularization for acute lower extremity ischemia. J. Vasc. Surg..

[20] Plate G., Oredsson S., Lanke J. (2009). When is thrombolysis for acute lower limb ischemia worthwhile?. Eur. J. Vasc. Endovasc. Surg..

[21] Nackman G.B., Walsh D.B., Fillinger M.F. (1997). Thrombolysis of occluded infrainguinal vein grafts: predictors of outcome. J. Vasc. Surg..

[22] Bhatnagar A., Whitsel L.P., Ribisl K.M. (2014). Electronic cigarettes: a policy statement from the American Heart Association. Circulation.

[23] Clair C., Rigotti N.A., Porneala B. (2013). Association of smoking cessation and weight change with cardiovascular disease among adults with and without diabetes. JAMA.

[24] Earnshaw J.J., Whitman B., Foy C. (2004). National audit of thrombolysis for acute leg ischemia (NATALI): clinical factors associated with early outcome. J. Vasc. Surg..

[25] Kuoppala M., Franzen S., Lindblad B. (2008). Long-term prognostic factors after thrombolysis for lower limb ischemia. J. Vasc. Surg..

[26] Vakhitov D., Suominen V., Korhonen J. (2014). Independent factors predicting early lower limb intra-arterial thrombolysis failure. Ann. Vasc. Surg..

[27] Lubitz S.A., Benjamin E.J., Ellinor P.T. (2010). Atrial fibrillation in congestive heart failure. Heart Fail. Clin..

[28] Zhou Q., Zhang Z., Ang X. (2021). A nomogram combined with radiomics features, albuminuria, and metabolic syndrome to predict the risk of myometrial invasion of bladder cancer. Transl. Cancer Res..

